# Chromosome-level genome assembly of largemouth bass (*Micropterus salmoides*) using PacBio and Hi-C technologies

**DOI:** 10.1038/s41597-022-01601-1

**Published:** 2022-08-06

**Authors:** Kuo He, Liulan Zhao, Zihao Yuan, Adelino Canario, Qiao Liu, Siyi Chen, Jiazhong Guo, Wei Luo, Haoxiao Yan, Dongmei Zhang, Lisen Li, Song Yang

**Affiliations:** 1 College of Animal Science and Technology, Sichuan Agricultural University, Chengdu, Sichuan 611130 China; 2 CAS Key Laboratory of Experimental Marine Biology, CAS Center for Ocean Mega-Science, Institute of Oceanology, Chinese Academy of Sciences, Qingdao, 266071 China; 3 Laboratory for Marine Biology and Biotechnology, Qingdao National Laboratory for Marine Science and Technology, Qingdao, 266071 China; 4 Comparative Endocrinology and Integrative Biology, Centre of Marine Sciences, Universidade Do Algarve, Campus de Gambelas, 8005-139 Faro, Portugal

**Keywords:** Genome, Agricultural genetics

## Abstract

The largemouth bass (*Micropterus salmoides*) has become a cosmopolitan species due to its widespread introduction as game or domesticated fish. Here a high-quality chromosome-level reference genome of *M. salmoides* was produced by combining Illumina paired-end sequencing, PacBio single molecule sequencing technique (SMRT) and High-through chromosome conformation capture (Hi-C) technologies. Ultimately, the genome was assembled into 844.88 Mb with a contig N50 of 15.68 Mb and scaffold N50 length of 35.77 Mb. About 99.9% assembly genome sequences (844.00 Mb) could be anchored to 23 chromosomes, and 98.03% assembly genome sequences could be ordered and directed. The genome contained 38.19% repeat sequences and 2693 noncoding RNAs. A total of 26,370 protein-coding genes from 3415 gene families were predicted, of which 97.69% were functionally annotated. The high-quality genome assembly will be a fundamental resource to study and understand how *M. salmoides* adapt to novel and changing environments around the world, and also be expected to contribute to the genetic breeding and other research.

## Abstract (summary)

**Table:** 

Measurement(s)	genome assemble
Technology Type(s)	Illumina paired-end sequencing, PacBio single molecule sequencing technique (SMRT) and High-through chromosome conformation capture (Hi-C) technologies
Sample Characteristic - Organism	Micropterus floridanus

## Background & Summary

The largemouth bass, *Micropterus salmoides* (Perciformes, Centrarchidae), is a native of North America introduced in other parts of the world, including the Iberian Peninsula, Italy, Mexico and China, either as a game or farmed fish^1–3^. It is now one of the top ten most common aquatic species in every continent, except Antarctica^4,5^, and has been listed among the top 100 invasive species^6^, with temperature and hydrologic changes as main predictors of its distribution^1,7^. Although its main habitat is freshwater lakes and rivers, it colonizes brackish waters, such as in the Gulf of Mexico and the Atlantic coasts of North America^8^. Largemouth bass has been introduced into China from the US in 1983^2^, and it has become one of the main aquaculture species in China for its fast growth^2,9^.

The whole genome information is the basis for studying the nature of organisms, including advantages during biological invasions and adaptation to extreme environments such as hypoxia^10–12^, climate change^13,14^, temperature^15,16^ and salinity^17,18^. With the development of sequencing technology, genome research has been studied more deeply and accurately^19^. More and more fish genomes have been decoded, such as yellow perch (*Perca flavescens*)^20^, golden pompano (*Trachinotus ovatus*)^21^ and dark sleeper (*Odontobutis potamophila*)^22^, etc. Moreover, the Nile tilapia and Pacific bluefin tuna genome have been re-sequenced to improve the genome assembly and fill the previously missed gaps^23,24^. These genome studies have greatly elevated our understanding about genetics, environmental adaptive selection, and evolutionary history of the target species. These more detailed genomic data can also facilitate studies on nutritional requirements, disease control and prevention, and to improve traits of economic interest^25–27^.

**Fig. 1:**
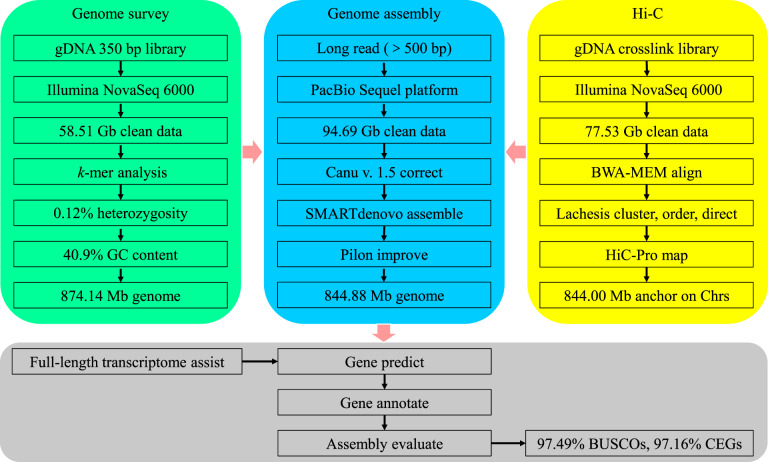
The pipelines overview of the largemouth bass chromosome-level genome assembly. Chrs: chromosomes.

In the present study, a novel high-quality chromosome-level genome assembly of largemouth bass was generated by single-molecule real-time sequencing combined with Illumina paired-end sequencing and Hi-C (Fig. 1). The final assembled genome size of *M. salmoides* was 844.88 Mb with an N50 contig length of 15.30 Mb and scaffold N50 length of 35.77 Mb. A total of 844.00 Mb assembled genome sequences were anchored on 23 chromosomes. The genome contained 38.19% repeat sequences and 2693 noncoding RNAs. A total of 26,370 protein-coding genes from 3415 gene families were predicted, of which 97.69% were functionally annotated.

## Methods

### Ethics statement

All experiments were performed according to the Guidelines for the Care and Use of Laboratory Animals in China. The sampled fish in this study was approved by the Institutional Animal Care and Use Committee (IACUC) of the College of Animal Science and Technology of Sichuan Agricultural University, Sichuan, China, under permit No. DKY-YS13287.

### Sequencing libraries

Tissues from a two-year-old adult female largemouth bass (body weight 1487 g, length 36 cm), obtained from an aquaculture farm of Chongzhou, Sichuan province, China, were used to construct genomic DNA sequencing libraries (muscle) and transcriptome sequencing libraries (liver, brain, muscle, heart, kidney, gill, and gonad). All the tissues were stored in liquid nitrogen until use.

For short-read sequencing, genomic DNA was extracted from 500 mg of muscle using cetyl trimethylammonium bromide (CTAB) before chloroform purification. The genomic DNA was sonicated to a fragment size of 350 bp and the paired-end genomic library was prepared following the Illumina standard protocol, including terminal repair, polyA and adaptor addition, target fragment selection and PCR processes (Illumina, San Diego, CA, USA). The resulted library was quality checked using Agilent Bioanalyser 2100 and qPCR, and sequenced on an Illumina NovaSeq 6000 sequencing platform with paired-end 150 bp read layout.

For long-read sequencing, genomic DNA (~8 µg) was sheared into a large fragment by g-TUBE (Covaris), purified and recovered by AMpure PB magnetic beads, and used to construct single-molecule real-time bell (SMRTbell) sequencing libraries by the SMRTbell Template Prep Kit 2.0 (PacBio)^28^. The end-repaired fragments were size-selected using the Blue Pippin Size-Selection System (Sage Science, MA, USA), and damage-repaired using the SMRTbell Damage Repair Kit (PacBio). Then the products were combined polymerase using the PacBio DNA/Polymerase Kit before sequenced on the PacBio Sequel platform.

The full-length transcriptome was used to generate RNA data for gene prediction from a sample pool consisting of muscle, liver, gonad, kidney, gut, blood, and gills. Total RNA was extracted by TRIzol extraction reagent (Invitrogen, USA) according to the manufacturer’s protocol. RNA purity was checked using the NanoPhotometer spectrophotometer (IMPLEN, CA, USA). RNA concentration was measured using Qubit RNA Assay Kit in Qubit 2.0 Flurometer (Life Technologies, CA, USA). Then, these tissues RNA were equally mixed to product cDNA using the SMARTer PCR cDNA Synthesis Kit and sequencing by one SMRT flow cell on the PacBio Sequel platform. Raw reads were processed into error corrected reads of insert (ROIs) using Iso-seq pipeline with minFullPass = 0 and minPredictedAccuracy = 0.90. Next, full-length, non-chemiric (FLNC) transcripts were determined by searching for the polyA tail signal and the 5′ and 3′ cDNA primers in ROIs. Full-length consensus sequences obtained from ICE (Iterative Clustering for Error Correction) were polished using Quiver. Finally, Full-length transcriptome sequencing yielded 20 Gb of clean data, including 26,369 high-quality consensus isoforms sequences with an average length of 2,895 bp.

### Genome survey and assembly

**Fig. 2:**
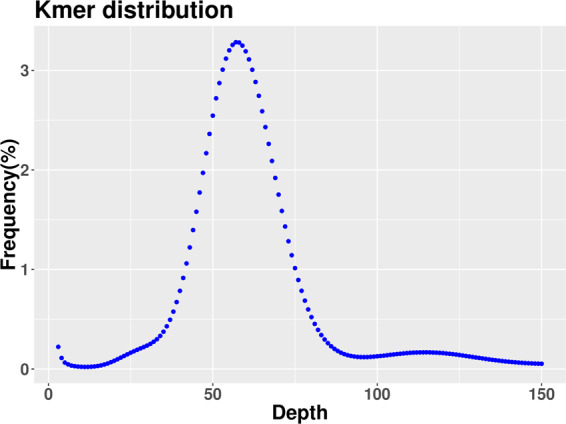
K‐mer distribution of *M. salmoides* genome sequencing reads. The K-mers distribution (K = 19) was constructed using 350 bp library data. A total of 49,157,214,151 K-mers were used for genomic length estimation after the removal of the K-mers with abnormal depth. The peak 19‐mer depth was 56, and the genome size was calculated as 49,157,214,151/56 = 874.14 Mb.

The size, heterozygosity, and repetitive sequences in the *M. salmoides* genome were estimated by the analysis of *k*-mer frequency distribution of Illumina paired-end reads using the kmer_freq_stat script (Biomarker Technologies, Beijing, China), based on the formula G = (N *k*-mer - Nerror_*k*-mer)/D (where G: genome size; N *k*-mer: the number of *k*-mers; Nerror_*k*-mer: the number of depth 1 *k*-mers; D: the *k*-mer depth). After removing the *k*-mers with abnormal depth, a total of 49.16 M *k*-mers were obtained with a *k*-mers peak at a depth of 56 (Fig. 2). A total of 58.51 Gb high-quality filtered data was generated from the Illumina short read DNA library, with 66.94 × genome coverage, a Q20 of 96.63% and a Q30 of 91.36% (Table 1). The genome size was estimated at 874.14 Mb, with 0.12% heterozygosity, 30.03% repetitive sequences, and 40.88% GC content (Table 1).

**Table 1:** *M. salmoides* genome sequencing statistics.

Library	Sequencing platform	Clean data (Gb)	Depth (×)	Contig N50 (Mb)	GC content (%)	Q20 (%)	Q30 (%)	Genome size (Mb)
Short reads	Illumina NovaSeq 6000	58.51	66.94	—	40.88	96.63	91.36	874.14
Long reads	PacBio Sequel	94.69	112.07	15.68	40.78	—	—	844.88
Hi-C	Illumina NovaSeq 6000	77.53	94.06	15.30	40.78	97.59	93.49	844.00

For long-read sequencing, reads longer than 500 bp generated by the PacBio Sequel platform were collected and a *de novo* genome was assembled initially using SMARTdenovo^29^ based on the data corrected by Canu v. 1.5^30^. Subsequently, three rounds of refinement of the *de novo* genome were performed using Pilon^31^ by Illumina short read sequencing data. Finally, the long-read SMRTbell library generated a total of 94.69 Gb (112.07 × genome coverage) with a reads N50 of 35.34 kb and an average read length of 24.75 kb. After error correction and assembly, an 844.88 Mb genome was assembled from 265 contigs with a N50 of 15.68 Mb (Table 1).

### Hi-C analysis and chromosome assembly

Hi-C libraries were prepared as previously reported^32,33^. Briefly, muscle tissue cells were fixed with formaldehyde to maintain the 3D structure of DNA in cells and the cells were digested using restriction endonuclease Hind III. Then, biotin-labeled bases were introduced using the DNA terminal repair mechanism. DNA (4 µg) was fragmented by a Covaris S220 focused-ultrasonicator (Gene Company Limited, Hong Kong) and 300–700 bp fragments were recovered. The DNA fragments containing interaction relationships were captured by streptavidin immunomagnetic beads for library construction. Library concentration and insert size were determined using the Qubit 3.0 and LabChip GX platforms (PerkinElmer), respectively. qPCR was used to estimate the effective concentration of the library. High quality Hi-C libraries were sequenced on the Illumina NovaSeq 6000 sequencing platform, and the sequencing data were used for chromosome-level assembly^34^. The software Burrows-Wheeler Aligner (BWA-MEM v. 0.7.10-r789) was used to align the sequencing pair-end clean reads with the sequence of the assembled genome to obtain the uniquely mapped read pairs^35^. The uniquely mapped read pairs were processed using HiC-Pro^36^. The genome contigs, split into 50 kb segments, combined with uniquely matched Hi-C data, were clustered, ordered and directed onto the pseudochromosomes using LACHESIS^34^ with the following parameters: CLUSTER_MIN_RE_SITES = 30; CLUSTER_MAX_LINK_DENSITY = 2; CLUSTER_NONINFORMATIVE_RATIO = 2; ORDER_MIN_N_RES_IN_TRUN = 68; ORD-ER_MIN_N_RES_I-N_SHREDS = 67. Finally, the chromosome assemblies were cut into 100 kb bins of equal lengths and the interaction signals generated by the valid mapped read pairs between each bin were visualized in a heat map.

**Fig. 3:**
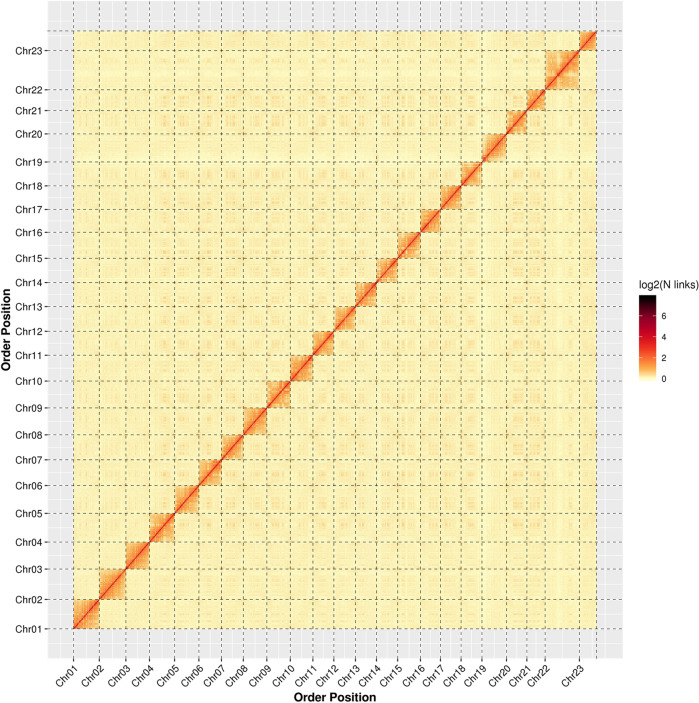
Hi-C assembly of chromosome interactive heat map. Chr01 - Chr23 are the abbreviations of 23 Chromosome. The abscissa and ordinate represent the order of each bin on the corresponding chromosome group. The colour block illuminates the intensity of interaction from yellow (low) to red (high).

In total, 277.88 million read pairs (77.53 Gb clean data; 94.06 × coverage of the genome) were generated from the Hi-C library (Table 1), of which 77.26% were uniquely mapped on the assembled genome. Of the unique mapped read pairs, 60.67% were the valid interaction pairs (130.26 million), which were used for the next Hi-C assembly (Table S1). A total of 844.00 Mb (99.9%) assembled genome sequences were anchored on 23 chromosomes, and the order and direction of 827.39 Mb (98.03%) sequences could be determined. The detailed distribution of each chromosome sequence was shown in Table 2. The heat map of the Hi-C assembly interaction bins is consistent a genome assembly of excellent quality (Fig. 3). Finally, the genome size of *M. salmoides* was assembled at 844.88 Mb, while contig N50 and scaffold N50 were 15.30 Mb and 35.77 Mb, respectively (Table 1).

**Table 2:** The sequence distribution of each chromosome using Hi-C technology.

Group	Cluster Num	Cluster Len	Order Num	Order Len
Chr01	5	40,821,207	4	40,732,462
Chr02	15	42,659,052	9	42,039,393
Chr03	7	37,588,897	6	37,343,944
Chr04	13	40,393,715	9	39,732,765
Chr05	15	39,747,164	6	38,411,921
Chr06	10	36,025,099	6	35,600,334
Chr07	9	34,881,373	6	34,516,066
Chr08	2	37,271,896	2	37,271,896
Chr09	5	37,188,422	4	37,114,295
Chr10	4	36,011,566	3	35,768,921
Chr11	11	33,902,165	5	33,113,071
Chr12	15	35,527,541	8	34,268,756
Chr13	5	33,494,735	4	33,265,410
Chr14	11	34,134,741	8	33,564,293
Chr15	24	37,902,394	11	35,937,762
Chr16	9	32,104,916	6	31,675,598
Chr17	7	32,964,910	4	32,674,911
Chr18	26	34,562,858	13	33,055,325
Chr19	31	41,218,652	16	38,871,204
Chr20	6	32,259,510	5	32,214,040
Chr21	3	28,886,792	3	28,886,792
Chr22	18	56,175,891	7	54,208,627
Chr23	15	28,271,698	8	27,127,050
Total (Ratio %)	266 (97.08)	843995194 (99.9)	153 (57.52)	827394836 (98.03)

Note: Chr01-23 represent 23 chromosomes; Cluster Num: the number of sequences located on a chromosome; Cluster Len: the length of sequence located on a chromosome; Order Num: the number of sequences of the direction can be determined; Order Len: the sequence length of the direction can be determined.

### Repeats prediction

The repetitive elements of the *M. salmoides* genome were identified and annotated using RepeatModeler2 containing RECON^37^ and RepeatScout^38^. The derived repetitive sequences were searched against curated libraries and the repetitive DNA element databases Repbase^39^, REXdb^40^ and Dfam^41^. The LTR retrotransposon retriever^42^ was applied to identify the output from LTRharvest^43^ and LTR_FINDER^44^. The results were combined and deduplicated, and the repetitive elements were finalized by RepeatMasker^45^. About 38.19% *M. salmoides* genome was repetitive sequences, composed mainly of class II transposable elements (Table 3).

**Table 3:** The repeat sequence statistics of assembled genome.

Type	Number	Length (bp)	Rate (%)
Class I: Retroelement	522983	121796357	14.42
DIRS	20880	6630621	0.78
LINE	234381	60698831	7.18
LTR/Caulimovirus	88	7962	0.00
LTR/Copia	12013	2607825	0.31
LTR/ERV	47996	6147308	0.73
LTR/Gypsy	103367	26357115	3.12
LTR/Ngaro	16775	2880533	0.34
LTR/Pao	13115	2271034	0.27
LTR/Unknown	37371	10223745	1.21
LTR/Viper	61	3732	0.00
SINE	36936	3967651	0.47
Class II: DNA transposon	1033511	198005683	23.44
Academ	1422	193616	0.02
CACTA	77241	10508494	1.24
Crypton	16505	2182911	0.26
Dada	7317	1072578	0.13
Ginger	4624	459506	0.05
Helitron	24163	10814282	1.28
IS3EU	3766	495816	0.06
Kolobok	31541	7047090	0.83
MITE	33	1774	0.00
Maverick	6547	1489673	0.18
Merlin	3162	472124	0.06
Mutator	6833	746138	0.09
Novosib	12838	1115265	0.13
P	15516	3779929	0.45
PIF-Harbinger	72041	15425212	1.83
PiggyBac	14753	2257482	0.27
Sola	7027	701813	0.08
Stowaway	1	57	0.00
Tc1-Mariner	115093	29999050	3.55
Unknown	128215	21996228	2.60
Zator	1443	282595	0.03
Zisupton	26754	4017294	0.48
hAT	456676	82946756	9.82
Satellite	4604	769122	0.09
Unknown	11211	2133201	0.25
Total	1572309	322704363	38.19

Note: Type: the type of repetitive sequence (Class I: retrotransposons; Class II: DNA transposon); Number: the number of repetitive sequences; Length: the total length of predicted repetitive sequences; Rate (%): the proportion of repetitive sequences in the total genome.

### Genes prediction and annotation

The prediction of the genome gene structure was based on three different strategies: *ab initio*-based, homolog-based, and unigene-based. Genscan^46^, Augustus v2.4^47^, GlimmerHMM v3.0.4^48^, GeneID v1.4^49^ and SNAP (version 2006-07-28)^50^ were used to perform *ab initio*-based prediction. GeMoMa v1.3.1^51,52^ was used for prediction based on homologous species. Hisat v2.0.4^53^ and Stringtie v1.2.3^54^ were used for assembly based on reference transcripts, and TransDecoder v2.0 and GeneMarkS -t v5.1^55^ were used for gene prediction. PASA v2.0.2^56^ was used to predict unigene sequences based on unreferenced assembly of full-length transcriptome data. Finally, EVM v1.1.1^57^ was used to integrate the prediction results obtained by the above three methods, and PASA v2.0.2 was used to modify the final gene models. A total of 26,370 protein-coding genes were predicted by integrating the prediction of *ab initio*, homology-based and RNA-seq strategies (Table S2), with average gene length of 14,483 bp, exon length of 2,601 bp, coding sequence of 1,724 bp and intron length of 11,882 bp (Table 4). Finally, 25,760 genes (97.69% of the total) were successfully annotated GO, KEGG, KOG, TrEMBL, and NR database (Table S3).

**Table 4:** The basic information statistics of assembled genome.

Item	Count
Gene Number	26,370
Gene Length (bp)	381,932,021
Average Gene Length (bp)	14,483.58
Exon Length (bp)	68,599,926
Average Exon Length (bp)	2,601.44
Exon Number	260,466
Average Exon Number	9.88
CDS Length (bp)	45,485,238
Average CDS Length (bp)	1,724.89
CDS Number	253,748
Average CDS Number	9.62
Intron Length (bp)	313,332,095
Average Intron Length (bp)	11,882.14
Intron Number	234,096
Average Intron Number	8.88

Blastn searches using the Rfam database^58^, as input against the *M. salmoides* genome was used to identify microRNA and rRNA and tRNAscan-SE^59^ was used to identify tRNA. Non-coding RNAs were predicted to be 2,639, including 633 microRNAs (miRNA) of 84 families, 230 rRNA genes of 4 families and 1,830 tRNA genes of 25 families (Table S4). Pseudogenes were predicted in the following way. The predicted protein sequences were used to search for homologous gene sequences (putative genes) through BLAT alignment^60^. Then GeneWise^61^ was used to search for immature termination codons and code-shifting mutations in the gene sequences to obtain pseudogenes. In total, 986 pseudogenes were identified with a total length of 5,885,501 bp and an average length of 5,969 bp (Table S4).

## Data Records

The sequencing data (Full-length transcriptome, Hi-C, Illumina and PacBio) have been deposited in SRA (Sequence Read Archive) database as SRR12886575^62^, SRR12886576^63^, SRR12886577^64^, and SRR12886578^65^. The assembly genome data was deposited in GenBank^66^. The assembly genome data, gene CDS and Exon data and functional annotations were also stored in Figshare^67^.

## Technical Validation

The assembly was evaluated using three criteria: the mapping of Illumina reads, core gene integrity, and BUSCO assessment. The Benchmarking Universal Single Copy Orthologs were searched in CEGMA v2.5^68^ and BUSCO v 3.0^69^ to evaluate the conserved core genes in the genome. The Illumina reads fully (99.54%) mapped to the assembled genome, including 97.78% of paired-end reads. A total of 445 out of in 458 conserved eukaryotic core genes from the CEGMA database were found in the assembled genome (Table S5). Finally, 97.49% of the complete BUSCOs were included in the assembled genome (Table S5). In summary, this is a high-quality *de novo* assembly reference genome.

## Supplementary information

SupplementaryTables

## Data Availability

All commands and pipelines used in data processing were executed according to the manual and protocols of the corresponding bioinformatics software.
