# Supplementary material for: Chromosome-level genome assembly of largemouth bass (Micropterus salmoides) using PacBio and Hi-C technologies
Source: Sci Data. 2022 Aug 6;9:482. doi: 10.1038/s41597-022-01601-1 (PMC9357066; doi:10.1038/s41597-022-01601-1)
Supplement: Supplementary file 1 — SupplementaryTables [file 41597_2022_1601_MOESM1_ESM.docx]

Supplemental Tables.

**Table S1. Statistics of Hi-C sequencing data of various types**

| Type | Number | Ratio (%) |
| --- | --- | --- |
| Unique Paired Alignments | 214,699,263 | 100 |
| Valid Interaction Pairs | 130,263,482 | 60.67 |
| Dangling End Pairs | 58,035,360 | 27.03 |
| Re-ligation Pairs | 8,231,089 | 3.83 |
| Self-cycle Pairs | 1,831,673 | 0.85 |
| Dumped Pairs | 16,337,659 | 7.61 |

**Unique Paired Alignments:** read pairs uniquely aligned to the genome; **Valid Interaction Pairs:** valid read pairs; **Dangling End Pairs:** read pairs of end dangling type in invalid data; **Re-ligation Pairs:** read pairs of adjacent connection type in invalid data; **Self-circle Ligation:** read pairs of self-connecting type in invalid data; **Dumped Pairs:** the other undefined read pairs in invalid data.

**Table S2. *M. salmoides* protein-coding gene prediction statistics**

| **Method** | **Software** | **Species** | **Gene number** |
| --- | --- | --- | --- |
| Ab initio | Genscan | - | 28,111 |
|  | Augustus | - | 32,705 |
|  | GlimmerHMM | - | 121,959 |
|  | GeneID | - | 23,539 |
|  | SNAP | - | 40,872 |
| Homology-based | GeMoMa | *Danio_rerio* | 24,479 |
|  |  | *Lates_calcarifer* | 23,979 |
|  |  | *Oreochromis_niloticus* | 26,475 |
|  |  | *Perca_flavescens* | 24,630 |
| RNAseq | TransDecoder | - | 63,249 |
|  | GeneMarkS-T | - | 41,564 |
|  | PASA | - | 47,812 |
| Integration | EVM | - | 26,370 |

**Table S3. Gene function annotation statistics of assembled genome**

| **Annotation** **database** | **Annotated number** | **Percentage (%)** |
| --- | --- | --- |
| GO | 12,704 | 48.18 |
| KEGG | 15,457 | 58.62 |
| KOG | 16,692 | 63.30 |
| TrEMBL | 25,485 | 96.64 |
| NR | 25,731 | 97.58 |
| All_Annotated | 25,760 | 97.69 |

**Table S4. Noncoding RNA and pseudogene statistics of assembled genome**

| **RNA classification** | **Number** | **Family** | **Pseudogene** | |
| --- | --- | --- | --- | --- |
| miRNA | 633 | 84 | Number | 986 |
| rRNA | 230 | 4 | Total length | 5,885,501 |
| tRNA | 1,830 | 25 | Average length | 5,969.07 |

**Table S5. Assembly result evaluation**

| CEGMA v2.5 | | BUSCO v2.0 | |
| --- | --- | --- | --- |
| Number of 458 CEG* present in assembly | 445 | Complete BUSCOs(C) | 4469 (97.49%) |
| 458 CEGs present in assemblies | 97.16% | Complete and single-copy BUSCOs(S) | 4356 (95.03%) |
| Number of 248 highly conserved CEGs present | 215 | Complete and duplicated BUSCOs(D) | 113 (2.47%) |
| 248 highly conserved CEGs present | 86.69% | Fragmented BUSCOs(F) | 43 (0.94%) |
| Total Lineage CEGMA | 458 | Total Lineage BUSCOs | 4,584 |

**458 CEGs* (Total Lineage CEGMA):** 458 conserved core genes of eukaryotes in CEGMA v2.5 database; **Number of 458 CEGs* present in assembly:** the number of genes in CEGMA v2.5 database found in the assembled genome; **Number of 248 highly conserved CEGs present:** the number of genes in the 248 highly conserved CEGs found in the assembled genome. **Total Lineage BUSCOs:** 4,584 conserved core genes contained in the BUSCO v2 database; **Complete BUSCOs(C):** the number of complete genes found in assembled genome; **Complete and single-copy BUSCOs(S):** the number of single-copy genes found in the assembled genome; **Complete and duplicated BUSCOs(D):** the number of multicopy gene number found in the assembled genome; **Fragmented BUSCOs(F):** the number of incomplete gene number found in the assembled genome.
